# Preliminary Evidence of Motor Outcomes in Children with Autism Spectrum Disorder Following Equine-Assisted Therapy and Dual-Task Training: A Pilot Study

**DOI:** 10.3390/sports14050190

**Published:** 2026-05-06

**Authors:** Carlo della Valle, Giulia Di Martino, Alessio Melis, Lorenzo Persichini, Chiara De Santis Del Tavano, Claudia Cerulli, Giovanni Fiorilli, Giuseppe Calcagno, Enzo Iuliano, Alessandra di Cagno

**Affiliations:** 1 Department of Neurosciences, Biomedicine and Movement, University of Verona, 37314 Verona, Italy; carlo.dellavalle@univr.it; 2 Department of Medicine and Health Sciences, University of Molise, 86100 Campobasso, Italy; a.melis@studenti.unimol.it (A.M.); fiorilli@unimol.it (G.F.); giuseppe.calcagno@unimol.it (G.C.); 3 Department of Human Sciences, Guglielmo Marconi University, 00193 Rome, Italy; g.dimartino@unimarconi.it; 4 Department of Movement, Human and Health Sciences, University of Rome “Foro Italico”, 00135 Rome, Italy; l.persichini1@studenti.uniroma4.it (L.P.); claudia.cerulli@uniroma4.it (C.C.); 5 ASD Sociale “Il Giardino di Filippo-Agriland”, 01100 Viterbo, Italy; chiafilippo@gmail.com; 6 Department of Theoretical and Applied Sciences, eCampus University, 22060 Novedrate, Italy; enzo.iuliano@ecampus.it

**Keywords:** hippotherapy, cognitive–motor intervention, motor skills, autism, executive function, physical activity

## Abstract

Background: Equine-assisted therapy (EAT) is a supportive intervention for children with autism spectrum disorder (ASD). Aim: This pilot study evaluated the feasibility of a cognitive–motor EAT intervention to explore its preliminary effects on motor coordination in children with ASD. Methods: A single-group pre-test–post-test design was adopted. Twelve children (mean age: 10.08 ± 1.51 years; ASD level 1–2) participated in a 16-week EAT program. The intervention integrated EAT with cognitive dual-task activities targeting attention and perceptual processing. Motor performance was assessed before and after the intervention using the Movement Assessment Battery for Children (MABC-2). Results: The RM-ANOVA examined pre–post changes. A significant improvement in the MABC-2 Total Test Score was observed (*p* = 0.036; ηp^2^ = 0.34; 95% CI [0.541, 13.792]), indicating a trend of improvement of global motor coordination. No significant improvements were observed in Manual Dexterity, Aiming and Catching, and Balance subscale scores. Conclusions: A cognitively enriched EAT program promoted improvements in overall motor competence in ASD children, even without measurable changes in specific motor skills. The intervention was feasible and well-tolerated, with high adherence and no adverse events. These preliminary findings support the feasibility and potential value of integrating cognitive–motor demands into EAT and provide a rationale for larger randomized controlled studies.

## 1. Introduction

Recent evidence has highlighted a growing interest in equine-assisted therapies (EAT) as supportive interventions for children with autism spectrum disorder (ASD). These interventions utilize the horse’s movement and the equine environment as therapeutic modalities and have been associated with positive outcomes across multiple domains, including physical, social, behavioral, emotional, sensory, and cognitive functioning [1]. Qualitative investigations have further reported perceived improvements in well-being and resilience among participants and their families [2]. With respect to social functioning, EAT has been associated with significant improvements in social communication and social cognition, along with trends toward enhanced social awareness and social motivation [3,4]. One proposed mechanism underlying these effects is enhanced self-regulation, which has been identified as a key mediator of improvements in social functioning among ASD children [5]. Recent evidence also suggests that clinical severity of ASD may moderate intervention outcomes and that longitudinal follow-up is essential to determine the persistence of treatment effects [6,7]. Motor outcomes associated with EAT include improvements in coordination, strength, balance, posture, and various aspects of motor competence in children with ASD [8]. Both short- and long-term experimental studies have confirmed benefits for postural control and functional motor skills, supporting the role of EAT as an effective complement to rehabilitation strategies [9,10]. Moreover, integrative approaches combining equine-assisted interventions with occupational therapy have demonstrated enhanced functional outcomes in real-world contexts [11,12].

Cognitive–motor dual-task (CMDT) training, which involves the simultaneous execution of motor and cognitive tasks, has shown promise in enhancing both motor and cognitive competencies across clinical and developmental populations [13]. A recent systematic review and meta-analysis indicates that structured combined cognitive and motor interventions significantly improve motor performance in children with ASD, often leading to reductions in core symptoms such as social deficits and repetitive behaviors [14].

Therefore, integrating CMDT into EAT may represent a targeted solution to overcome the potential limitations of EAT, providing the necessary cognitive load to optimize functional outcomes.

Based on this evidence, a critical knowledge gap remains regarding the optimal integration of cognitive and motor challenges within equine environments. The present study aimed to explore whether a 16-week cognitive–motor intervention implemented in an equine-assisted context could integrate the established therapeutic effects of EAT with the cognitive–motor benefits associated with dual-task training in children with autism spectrum disorder. The primary objective of this pilot study was to evaluate the feasibility of the intervention and to identify preliminary motor outcomes that could inform the design of a larger, controlled trial aimed at determining the effectiveness of EAT combined with dual-task training in improving difficulties in this population. A secondary aim was to contribute to the development of preliminary guidelines for optimizing EAT-CMDT protocols by identifying key features related to cognitive, motor, and social task progression in interventions for children with ASD.

## 2. Materials and Methods

### 2.1. Study Design

This pilot study employed a single-group pre-test–post-test design. A cohort of 12 children with ASD (level 1 and 2) underwent assessment before and after a 16-week EAT-based intervention administered once per week, combined with structured cognitive tasks (CMDT), targeting number, color, and movement-direction recognition, to evaluate whether this new type of intervention was associated with changes in motor functioning. The intervention was delivered on an individual basis, with each child receiving one-to-one sessions personalized to their specific abilities and needs, under the supervision of trained professionals. Task difficulty was systematically increased over time based on each child’s performance and developmental progression.

### 2.2. Participants

For the present study, twelve children of both sexes (mean age: 10.08 ± 1.51 years) were recruited. All participants had received a diagnosis of high-functioning autism from a child psychiatrist and were classified as autism level 1 or 2. Participants were enrolled and selected at the therapeutic riding center and social farm “Il Giardino di Filippo—Agriland” (Viterbo, Italy).

Inclusion criteria were as follows: (a) age between 9 and 13 years; (b) a clinical diagnosis of Autism Spectrum Disorder requiring level 1 or level 2 support, as defined by Diagnostic and Statistical Manual of Mental Disorders, Fifth Edition (DSM-5) criteria, confirmed by participants’ medical records; and (c) eligibility to participate in physical activity protocols, verified through clinical assessment. Exclusion criteria included the following: (a) use of medications or substances that could affect cognitive, executive, or motor functions; (b) the presence of contraindications for activities included in the protocol; and (c) recent injuries or medical conditions that would preclude participation in physical exercise. The study was conducted at the University of Rome “Foro Italico” with approval from the University Research Ethics Committee (CAR 1702023), on 19 September 2023, and in full compliance with the Declaration of Helsinki. The parents or legal guardians of the participants signed a guardian-informed consent form before the children’s participation.

### 2.3. Procedures

#### 2.3.1. Pre and Post-Intervention Assessment

Motor skills were assessed using the Movement Assessment Battery for Children—Second Edition (MABC-2) [15], a validated and age-normed tool for identifying and classifying motor difficulties in children and adolescents aged 3 to 16 years. The battery includes eight tasks grouped into three domains: Manual Dexterity (MD) (e.g., hand coordination), Aiming and Catching (A&C) (fine and gross motor precision), and Balance (BAL) (static and dynamic balance). Each domain was assessed independently, and raw scores from each task were converted into standard scores and combined into a total score. Based on normative data, the MABC-2 classifies motor performance using a three-level traffic light system: the red zone (total score < 56) indicates significant motor difficulties; the amber zone (total score 57–66) indicates a risk of motor difficulties; and the green zone (total score ≥ 67) corresponds to motor performance within the typical range.

#### 2.3.2. Equine-Assisted Therapy Protocol (EAT) Combined with Cognitive–Motor Dual-Task Training (CMDT)

The EAT was conducted at a therapeutic riding center. All participants received one 60 min therapeutic riding session per week, over a 16-week period. Prior to each session, children spent approximately 15 min interacting with the horse, including preparation and grooming activities designed to facilitate familiarity and engagement. Each session involved the child, a trained horse, a therapist/psychologist specialized in equestrian rehabilitation, and a kinesiologist.

The methodological framework of the EAT protocol was grounded in three key factors hypothesized to underlie its therapeutic effects: (a) the sensorimotor and embodied experience of riding the horse; (b) the specific movements and rhythmic patterns generated by the horse; and (c) the relational and behavioral characteristics of the horse itself.

The EAT sessions were combined with CMDT. The motor component consisted of structured equine-assisted activities (e.g., mounted riding, leading the horse, or postural tasks performed on the horse), which required continuous postural control, balance, and motor coordination. Simultaneously, the cognitive component involved structured tasks targeting the recognition of colors and geometric shapes, as well as phonological processing through letter–word association and assonance-based matching exercises. Cognitive stimuli were presented verbally or visually by the therapist during the execution of the motor task, requiring the child to identify, name, or match specific colours, shapes, or letter–word pairs while maintaining engagement in the EAT. Task difficulty was progressively increased according to a predefined progression within the standardized protocol based on individual performance, by varying the complexity of cognitive stimuli and the motor demands of the equine-assisted task. To guarantee a highly standardized and consistent intervention, the study trainer followed a rigorous operational protocol. Furthermore, to systematically monitor the implementation of the sessions and the children’s participation, all activities were recorded in a daily session log completed by the professionals involved in the intervention. This tool enabled structured documentation of participants’ attendance, the activities performed during each session, and adherence to the protocol throughout the entire intervention period. Participant adherence was high (≤1 missed session per child over 16 weeks), strongly indicating the protocol’s feasibility. Each therapeutic riding session was structured into seven sequential phases, as reported in Table 1.

Each phase included targeted exercises that were systematically repeated and reintroduced in varied and creative forms to promote learning consolidation. The use of materials with evocative and multisensory characteristics further supported engagement and skill acquisition.

### 2.4. Statistical Analysis

To evaluate whether children with ASD showed improvements in MABC-2 as a result of the intervention, an RM-MANOVA was conducted. The assumption of sphericity and normality of data (Shapiro–Wilk test) was tested before performing the RM-MANOVA. The analysis was performed using the 4 sub-scales of the MABC-2 as dependent variables (MD, A and C, BAL standard scores, and TTS). The pre- and post-intervention scores were used as repeated measures of the analysis (within factor). If the RM-MANOVA yields significant results, follow-up univariate analyses will be independently performed for MD, A&C, BAL standard scores, and TTS.

For all statistical analyses, the threshold for significance was set at *p* = 0.05. Partial eta-squared (ηp^2^) values were calculated to estimate effect sizes. All analyses were performed using SPSS Statistics, version 26 (IBM Corp., Armonk, NY, USA).

## 3. Results

The results of the RM-MANOVA indicated a significant Time effect (F_4,8_ = 5.832; *p* = 0.017; ηp^2^ = 0.745). Consequently, univariate analysis on MD, A&C, BAL standard scores, and TTS was performed.

MD standard scores did not show a significant improvement following the EAT intervention (F_1,11_ = 0.943; *p* = 0.352; ηp^2^ = 0.079). Similarly, no significant improvement was observed for the A&C standard scores after the intervention (F_1,11_ = 3.716; *p* = 0.080; ηp^2^ = 0.253). The BAL standard score showed a non-significant decline in post-intervention compared with pre-intervention values (F_1,11_ = 2.099; *p* = 0.175; ηp^2^ = 0.160).

In contrast, a significant effect emerged for the TTS. When the subdomains were considered collectively, children with ASD demonstrated a significant improvement from pre- to post-intervention assessments (F_1,11_ = 5.668; *p* = 0.036; ηp^2^ = 0.340).

**Figure 1:**
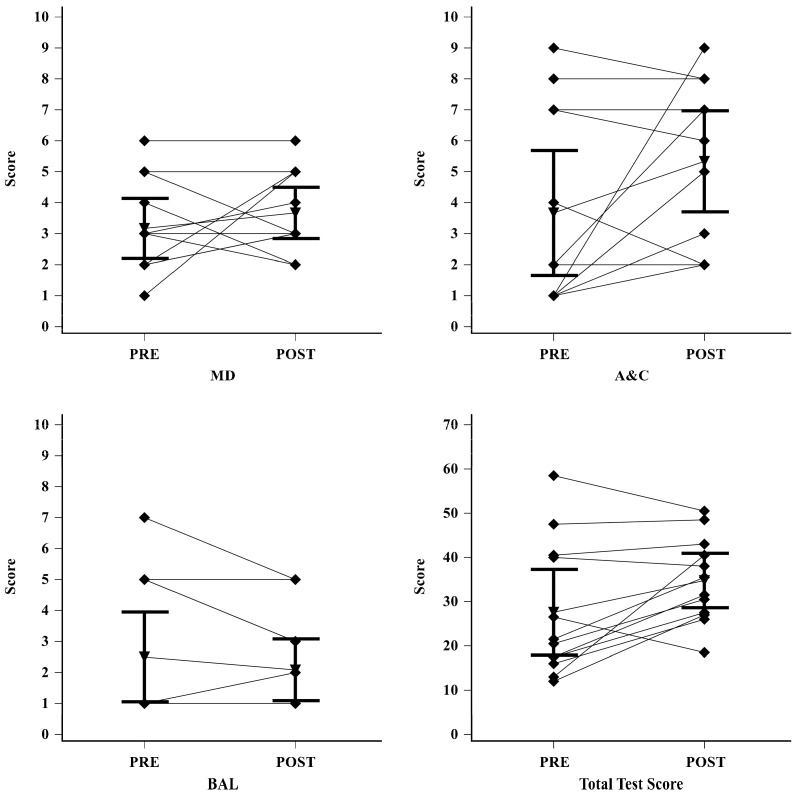
Results of pre- and post-intervention assessments. The plots present the results obtained by the participant group during the pre-test and post-test phases. Inverted triangles denote the mean values achieved by the participants, while the error bars indicate the 95% confidence interval (CI). Individual scores for each participant are represented by black diamonds. The following abbreviations are utilized: MD = Manual Dexterity standard score, A&C = Aiming and Catching standard score, and BAL = Balance standard score.

Detailed pre- and post-intervention scores for all outcome measures are reported in Table 2 and Figure 1.

## 4. Discussion

Although the results of this study provide preliminary evidence supporting the feasibility and efficacy of EAT-CMDT, improvements in global motor organization were not associated with improvements in specific motor skills as assessed with the MABC-2 test.

The significant increase in the Total Test Score likely reflects improvements in overall motor organization and multisensory integration capacity, supporting the notion that cognitively engaging interventions may be associated with improvements in global motor competence. Wang et al. [16] and Liang et al. [17] suggested that interventions requiring simultaneous processing of motor and cognitive tasks might be associated with enhanced neural efficiency within front-cerebellar networks. Additionally, the structured and closely monitored guidance provided throughout the training sessions likely facilitated sustained engagement, supported progressive and systematic error correction, and helped preserve participant motivation, all of which are recognized as key factors in promoting favorable intervention outcomes [18,19]. Nevertheless, the observed gains in overall motor coordination may be consistent with enhanced engagement of both lower- and higher-level executive functions, as proposed in previous research [20].

Despite the statistically significant improvements observed in the Total Test Score, it is crucial to note that all participants remained within the “red zone” of the MABC-2 classification (total score ≤ 56) at post-intervention. This discrepancy suggests that, although the EAT-CMDT protocol promoted a positive developmental trajectory in motor functioning, participants continued to maintain clinically significant motor difficulties. Although the 16-week intervention showed initial significant improvements in global motor coordination, all participants remained within the “red zone” (significant movement difficulty) of the MABC-2, without transitioning to the “amber zone” (at risk). This finding is not unexpected, as an intervention delivered once per week over 16 weeks results in a relatively limited cumulative dose, which is likely insufficient to produce changes in the diagnostic categories of motor classification. Therefore, while the combined protocol appears to promote initial improvements in motor competence, a progressive and longer-term intervention with increased training frequency may promote further gains and potentially enable a transition from the red zone to the amber zone within the MABC-2 classification.

The lack of improvement in Manual Dexterity and Aiming and Catching supports the principle of training specificity. As highlighted by Ruggeri et al. [21], motor skills in individuals with ASD are highly context-dependent. Therefore, the gross motor benefits do not automatically transfer to fine motor domains without explicit, high-repetition instruction [22].

Although not statistically significant, the observed worsening in balance scores may be indicative of transitional phases in motor learning, since EAT primarily targets seated, dynamic balance, reactive postural control, which is different from the standing static and dynamic tasks assessed in standard batteries [9].

Nevertheless, the results of this study should be interpreted with caution, as the frequency, duration, and physiological intensity of the EAT intervention may have influenced the observed outcomes. Specifically, this protocol was delivered once weekly, a frequency that may have limited the degree of motor and cognitive adaptations. Moreover, the intrinsic nature of EAT for young children with ASD may have involved relatively low metabolic intensity, potentially providing insufficient physiological stimulation to accelerate consolidation processes, despite the high level of motor–cognitive engagement [19]. This limitation becomes evident when compared with more effective CMDT interventions, which typically employ higher training frequencies and intensities, at least twice a week over a three-month period, to elicit significant improvements in specific motor skills [23]. Engagement in moderate-to-vigorous physical activity is a key determinant in optimizing cognitive and executive benefits in children with ASD [17].

Furthermore, this investigation should be considered a pilot study that provides a preliminary empirical basis for future research. The significant improvement in global motor organization offers a promising rationale for the potential efficacy of CMDT combined with EAT in this population. However, to translate these preliminary global benefits into consolidated specific skills, an optimization of the key intervention parameters, including training frequency, duration, and intensity, is crucial for maximizing adaptation [16,24,25].

This study has several limitations that should be acknowledged. First, as a pilot study, the limited sample size restricts the statistical power of the analyses and the generalizability of the findings. No a priori sample size was performed as convenience sampling was used in this study, and the low numerosity of the sample does not allow to exclude Type I and/or Type II errors. Furthermore, without formal corrections for multiple comparisons, the results should be considered exploratory and interpreted with caution due to the increased risk of Type I error.

Second, the absence of a sedentary control group or an active comparison group limits the ability to attribute the observed improvements specifically to the EAT–CMDT intervention. Compounding this limitation is the lack of assessor blinding. In a pre–post design lacking a control group, unblinded evaluations introduce a potential expectation bias, as the assessors may inadvertently anticipate positive outcomes. Future investigations must address this methodological gap by employing independent blinded assessors or by utilizing video-recorded assessments evaluated by external raters.

Third, the frequency, duration, and physiological intensity of the intervention may have resulted in a limited cumulative therapeutic dose. Over a 16-week period, it is plausible that changes in pediatric ASD populations may be influenced by confounding factors such as developmental maturation, concurrent routine therapies.

Finally, the exclusive reliance on a single outcome instrument (the MABC-2), lacking broader ecological and functional indicators of change, restricts the scope of interpretation regarding the transfer of these motor skills to daily life; future large-scale studies should incorporate functional and participation-level measures (e.g., daily living skills or school functioning) to better capture the broader clinical relevance of the combined intervention.

A key strength of this study is the development of an original CMDT protocol integrated within an EAT framework. This innovative intervention provides a structured and replicable approach that can be implemented and systematically tested in future randomized controlled trials involving larger samples, supporting the evaluation of its efficacy and clinical applicability.

## 5. Conclusions

This pilot study investigated the feasibility and preliminary motor outcomes of a cognitive–motor equine-assisted therapy intervention in children with autism spectrum disorder. The findings indicate that a 16-week horseback-based intervention is feasible and well tolerated in this population and may be associated with significant improvements in global motor organization, as reflected by the MABC-2 Total Test Score. Conversely, no significant changes were observed in specific motor subdomains, supporting the principle of task-specific motor learning. Although preliminary, the present findings provide a rationale for further investigation. Future randomized controlled trials with larger samples, increased training frequency, and higher intervention intensity are needed to confirm the effectiveness of EAT-CMDT.

## Figures and Tables

**Table 1:** Structure and aims of equine-assisted therapy (EAT) activities.

Activities	Aims
Horse care and grooming	Emotional regulation, sensory integration, and the development of a positive relational bond with the horse, while enhancing fine motor skills and attention.
Leading the horse from the ground along motor skill courses	Gross motor coordination, balance, spatial orientation, motor planning, and executive functioning.
Mounted horse-riding activities	Postural control, trunk stability, balance, combined with engagement of executive functions (attention, working memory, cognitive flexibility, and problem solving).
Horse-vaulting CMDT exercises	Dynamic balance, strength, and body awareness, confidence and self-efficacy.
Unsaddling, grooming, and stable-related activities	Autonomy, sequencing abilities, fine motor coordination, and executive functions.
Preparation of food for the horses	Cognitive skills, such as planning, categorization, and sequencing, and adherence to structured routines.
Feeding the horse	Social–emotional engagement through interaction with the animal.

**Table 2:** Results of pre- and post-intervention assessments.

Subscales	Pre-Intervention Score	Post-Intervention Score	95% Confidence Interval for Difference
Manual Dexterity Standard Score (MD)	3.17 ± 1.53	3.67 ± 1.3	−0.633 to 1.633
Aiming and Catching Standard Score (A&C)	3.67 ± 3.17	5.33 ± 2.57	−0.236 to 3.570
Balance Standard Score (BAL)	2.5 ± 2.28	2.08 ± 1.56	−1.050 to 0.216
Total Test Score	27.58 ± 15.24	34.75 ± 9.68 *	0.541 to 13.792
Traffic Light Score	All participants in Red Zone	All participants in Red Zone	-

* = *p* < 0.05 compared with pre-intervention score.

## Data Availability

All data are contained within the manuscript.

## Abbreviations

The following abbreviations are used in this manuscript:

ASDs	Autism Spectrum Disorders
CMDT	Cognitive–Motor Dual-Task
EAT	Equine-Assisted Therapy
MABC-2	Movement Assessment Battery Children—Second Edition
MD	Manual Dexterity
A&C	Aiming and Catching
BAL	Balance
TTS	Total Test Score
RM-ANOVA	Repeated Measures ANOVA
